# Effects of *Cinnamomum camphora* coppice planting on soil fertility, microbial community structure and enzyme activity in subtropical China

**DOI:** 10.3389/fmicb.2023.1104077

**Published:** 2023-02-03

**Authors:** Luyuan Sun, Jie Zhang, Jiao Zhao, Xianghui Lu, Changlong Xiao, Zufei Xiao, Ting Zhang, Yueqi Gu, He Sun, Han Liu, Yanli Li

**Affiliations:** ^1^Jiangxi Provincial Engineering Research Center for Seed-Breeding and Utilization of Camphor Trees, School of Hydraulic and Ecological Engineering, Nanchang Institute of Technology, Nanchang, China; ^2^College of Agriculture, Yangtze University, Jingzhou, China; ^3^Jiangxi Academic of Forestry, Nanchang, China

**Keywords:** *Cinnamomum camphora* coppice planting, soil fertility, bacterial community structure, fungal community structure, enzyme activity

## Abstract

*Cinnamomum camphora* (*C. camphora*) is a broad-leaved evergreen tree cultivated in subtropical China. Currently, the use of *C. camphora* clonal cuttings for coppice management has become popular. However, the effects of *C. camphora* coppice planting on soil abiotic and biotic variances remained unclear. In this study, we collected soil from three points in the seven-year *C. camphora* coppice planting land: under the tree canopy (P15), between trees (P50), and abandoned land (Control) to investigate the effects of *C. camphora* coppice planting on soil fertility, microbial community structure and enzyme activity. The results revealed that *C. camphora* coppice planting significantly increased soil fertility in the point under the tree canopy (P15) and point between trees (P50), and P15 had more significant effects than P50. Meanwhile, in P15 and P50, soil bacterial, fungal alpha-diversity were improved and microbial community structures were also changed. And the changes of soil organic carbon and total nitrogen promote the transformation of soil bacterial, fungal community structures, respectively. In addition, *C. camphora* coppice planting significantly (*p* < 0.05) increased soil urease (UE), polyphenol oxidase, and peroxidase activities, while significantly decreased soil ACP activity. This study demonstrated that the *C. camphora* coppice planting could improve soil fertility in subtropical China, which promoted the transformation of soil microbial community from *oligotrophs* (*K*-strategist) to *copiotrophs* (*r*-strategist). Thus, this work can provide a theoretical basis for soil nutrient variation and productive management of *C. camphora* coppice plantation in subtropical China.

## Introduction

Tree planting has been proposed as a practical method to prevent soil degradation. It can increase vegetation coverage, reduce rain erosion and enhance soil water and fertilizer retention ability (Speranza et al., 2019; Kumar et al., 2021). Meanwhile, the litterfall will return generous amounts of organic matter to the soil and the well-developed roots can activate the soil nutrients elements (Asigbaase et al., 2021). All of these are vital in the formation and evolution of planting land soil fertility (Feng et al., 2019). Various studies proved that tree planting can improve the soil nutrient stocks and availabilities in the wasteland and farmland (Zarafshar et al., 2020). For instance, relative to the abandoned land, *Robinia pseudoacacia* planting significantly increased the soil total C, N, and P nutrients in Calcaric Cambisol of the Loess Plateau (Zhang et al., 2019). And *Shorea robusta* planting significantly enhanced the available N and P contents in the topsoil compared with the agricultural land used for *Oryza sativa* L. cultivation in tropical areas (Ahirwal and Maiti, 2016). Nevertheless, some researches declared that tree planting has the negative impact on soil fertility. Teng et al. (2020) summarized that the 19-year-old *Pinus sylvestris* var. *mongolica* planting decreased the soil organic carbon (SOC) and TN contents in aeolian sandy soil of northern China. Xu et al. (2021a) also recorded that in subtropical China, the *Manglietia glauca Blume* planting reduced the soil TN and AP contents relative to the abandoned land with 10.42 and 58.77%, respectively. Thus, soil fertility under tree planting may vary with tree species, soil types, and so on.

Soil microorganisms are highly sensitive to changes in soil nutrients and are regarded as one critical biological indicator of soil fertility (Carrara et al., 2018; Liu et al., 2018a; Sun et al., 2020). Tree planting will exert extensive and profound impacts on soil microorganisms (Thoms et al., 2010). A previous study recorded that 20-year-old *Ormosia hosiei* planting obviously improved the soil microbial (bacterial and fungal) biomass and diversity in red-yellow soil (Wan et al., 2021). While the rubber plantation caused the degradation of the soil microbial community structure relative to the natural forests in Latosols and Ferralsols in tropical regions (Monkai et al., 2018). The soil fertility variations caused by tree planting are the dominant factors that drive variations in soil microbial community (Pereira et al., 2019). Xu et al. (2021b) believed that the increase of SOC and TN in *R. pseudoacacia* planting improved the microbial biomass and nutrients metabolism capability. On the contrary, Wu et al. (2015) discovered that with the increase of stand age in *Pinus elliottii* planting, decreased soil fertility leads to the decline of total PLFA amounts and carbon metabolic capability. Hence, to verify the changes of soil microbial community and determine their driving factors after tree planting are conducive to timely and efficiently adjusting the management strategies, thereby maintaining the stability of the forest ecosystems and promoting the sustainable development of forest resources.

Soil enzymes are secreted by soil microorganisms, and they have a pivotal influence on biochemical processes such as litter decomposition and element cycling (Rosinger et al., 2019). Tree planting induces strong effects on soil enzyme activities, and their activities may vary among different tree species (Bueno de Mesquita et al., 2017). Chen et al. (2022) reported that the *Hippophae rhamnoides* (broadleaf) planting increased enzyme activities of soil catalase, urease, and polyphenol oxidase (PPO) compared with the *Larix gmelina* (conifer) planting. Wang et al. (2020) proved that broadleaved tree planting increased soil PPO and peroxidase (POD) activities, while coniferous tree planting increased soil acid phosphatase activity (Moghimian et al., 2017). Cause broadleaved trees have higher litterfall quantity and quality (with low carbon to nitrogen ratio), which can produce large amounts of easily decomposed organic matter to stimulate microbial reproduction and thus secrete more extracellular enzymes (Nakayama et al., 2019). On the contrary, coniferous trees have lower litterfall quantity and quality (with high carbon to nitrogen ratio) with a great deal of hardly decomposed tannin, resin, and wax, which is unfavorable for metabolism of soil microorganisms (Ushio et al., 2008). Therefore, effects on soil enzyme activities will influence by different tree species.

*Cinnamomum camphora* (L.) Presl is a broad-leaved evergreen tree belonging to the Lauraceae family, which is widely distributed in subtropical China (Liu et al., 2022). *C. camphora* tree can produce large amounts of secondary metabolites such as essential oil (Zhong et al., 2022). Traditionally, the whole *C. camphora* tree was cut down to extract essential oil from its roots, stems, and leaves, and this disposable production method will cause great damage to the ecological environment (Liu et al., 2018b). Currently, using the *C. camphora* clonal cuttings for coppice management has become popular in southern China (Zhao, 2021). From the second year after transplanting, its aerial parts are felled down at 20 cm away from the ground to extract essential oil during July to September every year. Then the remaining *C. camphora* tree stump will sprout year after year, so as to realize the circular production. This management method is similar to regular harvest of crops, which is fundamentally different from traditional tree planting. However, the effects of *C. camphora* coppice planting patterns on soil quality and its ecological effects are still unclear.

In this study, one 5-year-old camphor coppice plantation land was selected to compare with the adjacent abandoned land in subtropical China. We further chose the point under the tree canopy and the point between trees. The former is greatly affected by nutrients transfer and tree growth, while the latter is the opposite. The major objectives of the present study were: (1) to determine how the *C. camphora* coppice planting affected soil fertility. (2) To reveal how the soil microbial community structure varied with the *C. camphora* coppice planting, and confirm the key driving factors. (3) To explore how soil enzyme activity responded to the *C. camphora* coppice planting and clarify the main reasons. This study can offer a theoretical basis for scientifically guiding the production and management of *C. camphora* coppice plantations in subtropical China.

## Materials and methods

### Study area

The field experiment was conducted on *C. camphora* coppice plantation land in Guixi City, Jiangxi Province, southern China (28°17′46″ N, 117°13′28″E). This research region has the subtropical monsoon climate in which the mean annual temperature precipitation and sunlight hours are 18.8°C, 1980.8 mm, and 1611.5 h at this site, respectively. The soils derived from the quaternary red clay, was abandoned land before the experiment. The initial properties of the topsoil (0–15 cm) with soil pH of 4.83, and with SOC, total nitrogen (TN), available nitrogen (AN), available phosphorus (AP) of 12.95, 0.96, 102.93, 5.21 g·kg^−1^, respectively.

### Experiment design and soil sampling

The 5,000 m^2^ *C. camphora* coppice plantation land selected for this study was established in 2015. In early September of each year since 2016, the aboveground part of the *C. camphora* tree was felled at 20 cm away from the ground for essential oil extraction. The row and inter-plant spacings of the *C. camphora* tree are 1 m × 1 m, and the planting density is 10,000 tree·hm^−2^. Urea (contains 46% N), calcium magnesium phosphate (contains 12% P_2_O_5_) and potassium chloride (contains 60% K_2_O) were used for the nitrogen, phosphorus, potassium fertilizers respectively, and their application rate were all 150 g tree^−1^ year^−1^. 50% of the fertilizers were applied in early March and the remaining 50% were applied in late September after the *C. camphora* tree felling. The fertilization point (FP) was a 15 cm deep circle 25 cm from the center of the tree (Supplementary Figure S1).

In this study, we selected three different points as three treatments, each with four replicates: (1) Point under the tree canopy, 15 cm from the center of the tree (P15, inside the fertilization point); (2) point between trees, 50 cm from the center of the tree (P50, outside the fertilization point); (3) point in the abandoned land (control, next to the selected *C. camphora* coppice). The specific sampling scheme is shown (Supplementary Figure S1). Soil samples were randomly collected using the S-shaped sampling method after the *C. camphora* tree felling on September 12th, 2021. Each sample was carried out using a soil core sampler (diameter of 2.0 cm) from the depth of 0–15 cm. Each core was taken in 50 m^2^ areas, and twenty cores were mixed into one soil sample. Then pass the soils through the 2 mm sieve to remove the impurities and divided into three parts: one part was air-dried for soil fertility measurement, the second part was preserved at 4°C for enzyme activity measurement, and the remaining part was kept at −80°C for DNA extraction.

### Soil properties and enzyme activity

Soil pH value (soil to water ratio of 1:2.5) was measured with a pH meter (FE28-Standard, METTLER-TOLEDO, Switzerland; Sun et al., 2022). SOC was determined by dichromate oxidation and titration with ferrous sulfate. TN was determined using the Kjeldahl digestion distillation method. Available nitrogen (AN) and phosphorus (AP) were measured using alkali hydrolyzation and Bray method, respectively (Akhtar et al., 2018). Soil enzyme activities of invertase (INV), urease (UE), acid phosphatase (ACP), catalase (CAT), PPO, and POD were determined using kits from Beijing Solarbio Technology Co. Ltd. according to the test instructions (Tabatabai, 1994; Saiya-Cork et al., 2002).

### Soil microbial DNA extraction and Illumina MiSeq sequencing

DNA was extracted from half a gram of fresh soil using the FastDNA® SPIN Kit for soil. The purification of DNA was tested by Power Clean DNA Clean-Up Kit to remove PCR inhibitors. The eluted DNA was examined by 1% (m/v) agarose gel electrophoresis and quantified with NanoDrop® 2000 spectrophotometer. Aliquots of the DNA were stored in a −20°C freezer for subsequent analyses. Primer sets Eub338 and Eub518 were used to determine the bacterial biomass by quantitative real-time PCR (qPCR) on the ABI Prism® 7300 Real-Time detection system. Fungal abundance was determined by Primer set SSU_0817 and SSU_1196 of the 18S rRNA gene in the same method with bacteria. The V4-V5 highly variable regions of the bacterial 16S rRNA genes were amplified with barcoded universal primers 515F (5′-GTGCCAGCMGCCGCGGTAA-3′) and 907R (5′-CCGTCAATTCCTTTGAGTTT-3′; Biddle et al., 2008). And the ITS regions of the fungal rRNA genes were amplified by primer sets ITS1F (5’-CTTGGTCATTTAGAGGAAGTAA-3′) and ITS2R (5-GCTGCGTTCTTCATCGATGC-3′; Adams et al., 2013). The qPCR parameters were referred to Xiang et al. (2021) and Jin et al. (2021) methods.

### Bioinformatics analysis for raw sequences

The original raw sequences were analyzed using the QIIME v.1.91 pipeline (Caporaso et al., 2010). And short (<200 bp) and low quality (average scores <25) or sequences were removed before downstream analysis (Magoč and Salzberg, 2011). High-quality sequences at a 3% dissimilarity level were selected as OTUs using UNOISE algorithm (Edgar, 2016). Representative set sequences were analyzed by RDP classifier using the SILVA 132 database with a confidence threshold of 80% (Wang et al., 2007). The representative sequences of ITS were annotated for species using the UNITE database with the blast method (Kõljalg et al., 2013). For downstream analyses, the number of bacterial and fungal sequences was rarefied to 30, 772 and 36, 205 for each sample to uniform the sequencing depths, respectively. A total of 4,427 bacteria OTUs and 1,690 fungal OTUs were identified for subsequent analysis.

### Statistical analysis

Firstly, we distinguished whether the initial data were accord with the normal distribution. The non-normally distributed data were logarithm or square root-transformed to make them close to normal distribution. And the mean and standard deviations (e.g., soil fertility indexes and enzyme activities, microbial abundance, and alpha diversity indexes) of different treatments were calculated. One-way analysis of variance (ANOVA) by Duncan’s HSD test (*p* < 0.05) was used for multiple comparisons. Pearson correlation analysis was used to test the relationship between soil microbial abundance and alpha diversity indexes or enzyme activities and the soil fertility indexes. Constrained Principal of Coordinate Analysis (CAP) with Bray-Curtis distance was used to distinguish the differences of soil microbial communities or the enzyme profiling in different treatments, and analysis of similarity (ANOSIM) test was performed to verify whether the difference between treatments is significant. Using an Bray-Curtis distance matrix, the correlation between the soil microbial community structure or the enzyme profiling and soil fertility indexes was calculated using Mantel test with 999 permutations. The relationships between the soil microbial community composition or the enzyme profiling and soil fertility indexes were determined by redundancy analysis (RDA). Multivariate regression tree (MRT) was used to identify the key factors that shift the soil microbial community composition. Procrustes analysis was used to confirm the correlation between the soil enzyme profiling and microbial community composition in different treatments.

ANOVA and Pearson correlation analysis were conducted using the statistical software SPSS 20.0. ANOSIM, CAP, Mantel test, RDA, and Procrustes analysis were all done using the “vegan” package (Dixon, 2003), and MRT analysis was performed using the “mvpart” package of the R software (ver. 4.0.1; De’ath, 2003). The differences in soil microbial taxon and enzyme activities were assessed using STAMP software (Parks and Beiko, 2010).

## Results

### Soil fertility

Significant differences (*p* < 0.05) in soil fertility among different treatments were tested (Table 1). Compared with the Control, soil pH, SOC, TN, AN, and AP of the P15 were increased by 12.96% (*p* < 0.05), 44.29, 80.06% (*p* < 0.05), 79.46% (*p* < 0.05), and 560.16% (*p* < 0.05), respectively. Moreover, soil AN in P50 was significantly greater than Control with 33.99% (*p* < 0.05) increase, but there were no significant changes with other soil fertility indexes. Therefore, *C. camphora* coppice planting can increase soil fertility to a certain extent.

**Table 1:** Soil fertility in the *Cinnamomum camphora* coppice planting.

Variables	P15	P50	Control
pH	5.51 ± 0.68a	4.72 ± 0.17b	4.88 ± 0.10b
SOC (g kg^−1^)	19.56 ± 1.55a	12.60 ± 1.62b	13.55 ± 0.50ab
TN (g kg^−1^)	1.78 ± 0.21a	1.14 ± 0.11b	0.99 ± 0.04b
AN (g kg^−1^)	212.62 ± 24.96a	158.75 ± 18.79b	118.48 ± 9.60c
AP (g kg^−1^)	32.54 ± 6.42a	6.86 ± 0.65b	4.93 ± 0.54b

P15, point under the tree canopy; P50, point between trees; Control, point in the abandoned land; SOC, soil organic carbon; TN, total nitrogen; AN, alkaline nitrogen; AP, available phosphorus. Values (mean ± S.D.) in the same column followed by different letters are significantly different from Duncan’s HSD comparisons (*p* < 0.05).

### Soil bacterial and fungal community structure

**Figure 1:**
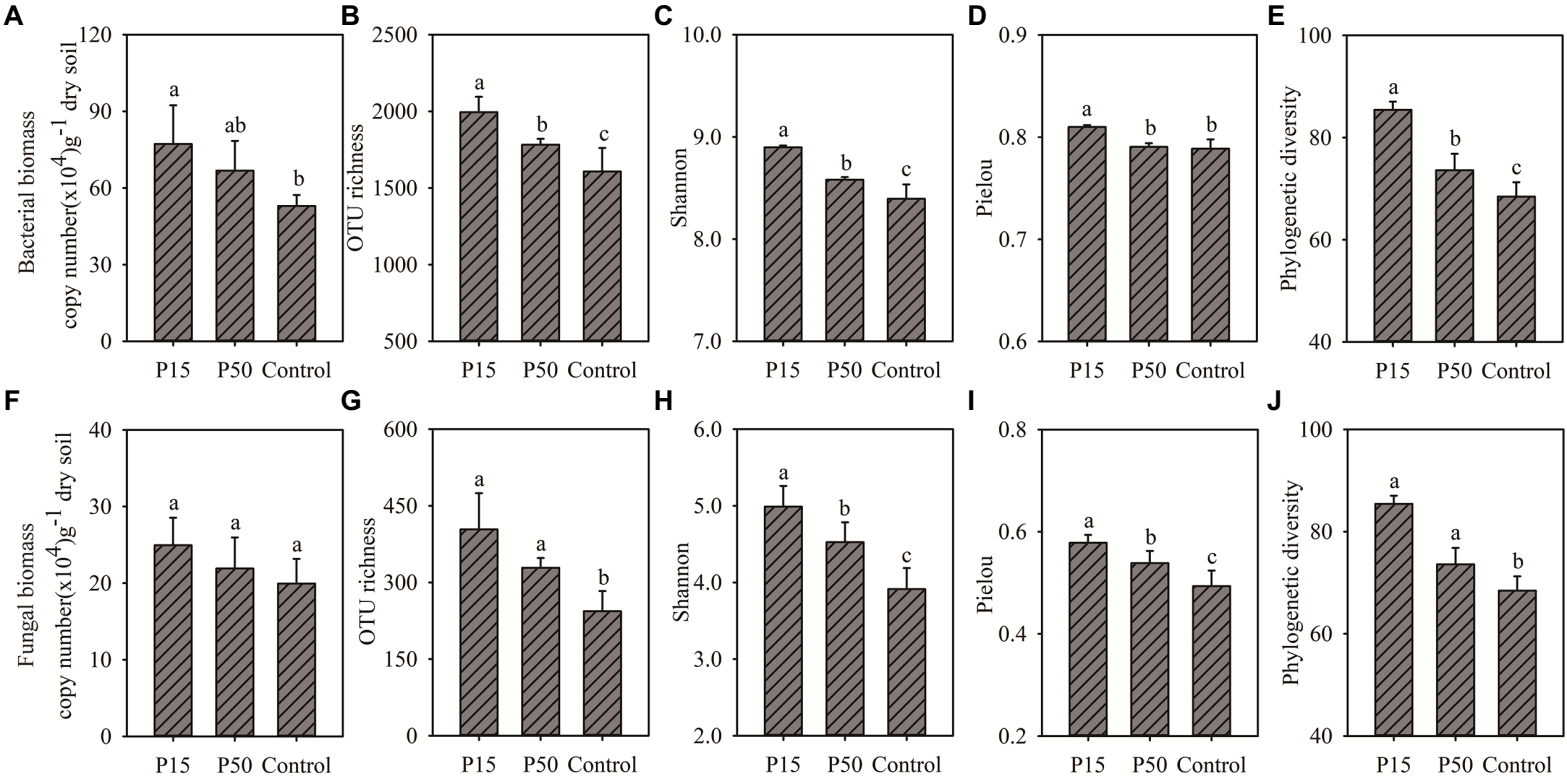
**(A)** Bacterial biomass, **(B)** Bacterial OTU richness, **(C)** Bacterial Shannon index, **(D)** Bacterial Pielou index, **(E)** Bacterial Phylogenetic diversity, **(F)** Fungal biomass, **(G)** Fungal OTU richness, **(H)** Fungal Shannon index, **(I)** Fungal Pielou index, **(J)** Fungal Phylogenetic diversity. Soil bacterial, fungal abundance and alpha-diversity under different treatments in the *Cinnamomum camphora* coppice planting. P15, point under the tree canopy; P50, point between trees; Control, point in the abandoned land. Bars represent mean; error bars denote standard deviation. Letters above bars represent differences from Duncan’s HSD comparisons (*p* < 0.05).

*Cinnamomum camphora* coppice planting can increase the soil microbial biomass and alpha-diversity (Figure 1). Soil bacterial biomass in P15 and P50 increased by 45.68% (*p* < 0.05) and 26.05% (*p* > 0.05) in comparison with Control, respectively (Figure 1A). The soil bacterial alpha-diversity in P15 and P50 were significantly higher relative to Control, except the pielou index in P50 (Figures 1B–D). Similarly, the P15 and P50 increased soil fungal biomass by 25.00 and 9.79% compared with the Control (Figure 1F). Furthermore, the four fungal alpha-diversity indexes increased significantly (*p* < 0.05) in P15 and P50 relative to the control (Figures 1G–J). The increase of bacterial or fungal biomass and diversity indexes in P15 was greater than that in P50.

For bacterial, in comparison with the Control, the P15 and P50 significantly (*p* < 0.05) improved relative abundance of Proteobacteria (from 29.35 to 40.95 and 36.82%, respectively), Bacteroidetes (from 1.30 to 5.33 and 5.68%, respectively) and Gemmatimonadetes (from 1.26 to 2.51 and 1.47%, respectively). Adversely, relative abundance of Chloroflexi in P15 and P50 was significantly (*p* < 0.05) decreased by 71.49 and 59.46% relative to the Control (Supplementary Figure S2). STAMP analysis was used to analyze significantly differentiated bacterial genera between treatments (Supplementary Figure S3). There were thirteen bacterial genera in P15 and six bacterial genera in P50 significant differential with Control (*p* < 0.05, effect size >1). Specifically, relative to Control, P15 reduced relative abundance of *Conexibacter*, *Ktedonobacter*, *Edaphobacter*, *Fimbriiglobus*, *Rhodanobacter* and so forth, but enhanced relative abundance of *Gaiella* and GP6. Similarly, in comparison with Control, the P50 decreased relative abundance of *Conexibacter* and *Ktedonobacter*, whereas increased relative abundance of *Flavitalea* and *Chryseolinea*.

For fungal, in comparison with Control, the P15 (*p* < 0.05) and P50 increased relative abundance of Zygomycota (by 61.31 and 34.70%) and Basidiomycota (by 149.59 and 36.60%), but reduced the relative abundance of Ascomycota by 35.37 and 18.54% (Supplementary Figure S2). Significantly differentiated fungal genera between treatments were also analyzed (Supplementary Figure S3). Correspondingly, four fungal genera in P15 and five fungal genera in P50 were significantly different with the Control (*p* < 0.05, effect size >1). The relative abundance of *Talaromyces* and *Hyaloscyphaceae* were reduced in both the P15 and P50 relative to Control. In addition, compared with Control, P15 increased relative abundance of *Westerdykella* and *Trichocomaceae*, whereas P50 enhanced relative abundance of *Fusarium*, *Archaeorhizomyces* and *Flagellospora*.

### Soil enzyme activities

*Cinnamomum camphora* coppice planting significantly affected the soil enzyme activities (Table 2). Soil UE, PPO, and POD activities were significant (*p* < 0.05) increased in P15 and P50 relative to Control, except the soil UE activity in the P50 (*p* > 0.05). Compared with Control, soil INV activity in P15 and P50 was increased (*p* > 0.05) by 47.98 and 37.58%. In addition, P15 and P50 significantly (*p* < 0.05) decreased the soil ACP activity, and P50 significantly (*p* < 0.05) decreased soil CAT activity in comparison with Control. STAMP analysis (*p* < 0.05, effect size >1) analyzed significantly different soil enzymes between treatments (Supplementary Figure S3). Specifically, in comparison with Control, P15 and P50 reduced the soil ACP activity, but enhanced the soil UE activity. Moreover, P15 also enhanced the soil POD activity.

**Table 2:** Soil enzyme activity in the *Cinnamomum camphora* coppice planting.

Variables	P15	P50	Control
INV (mg g^−1^ 24 h^−1^)	6.56 ± 1.97a	6.10 ± 1.30a	4.43 ± 0.66a
UE (μg g^−1^ 24 h^−1^)	342 ± 35a	247 ± 18b	212 ± 10b
ACP (μmol g^−1^ 24 h^−1^)	20.48 ± 1.85b	18.97 ± 1.49b	23.53 ± 0.81a
CAT (mg g^−1^ 24 h^−1^)	1.14 ± 0.07a	0.70 ± 0.08b	1.05 ± 0.03a
PPO (mg g^−1^ 24 h^−1^)	30.06 ± 4.24a	23.88 ± 0.64b	14.74 ± 3.90c
POD (mg g^−1^ 24 h^−1^)	25.36 ± 5.35a	16.58 ± 4.45b	2.68 ± 0.01c

P15, point under the tree canopy; P50, point between trees; Control, point in the abandoned land; INV, invertase; UE, urease; ACP, acid phosphatase; CAT, catalase; PPO, polyphenol oxidase; POD, peroxidase. Values (mean ± S.D.) in the same column followed by different letters are significantly different from Duncan’s HSD comparisons (*p* < 0.05).

### Correlations between soil microbial community composition or enzyme activities and soil fertility indexes

**Figure 2:**
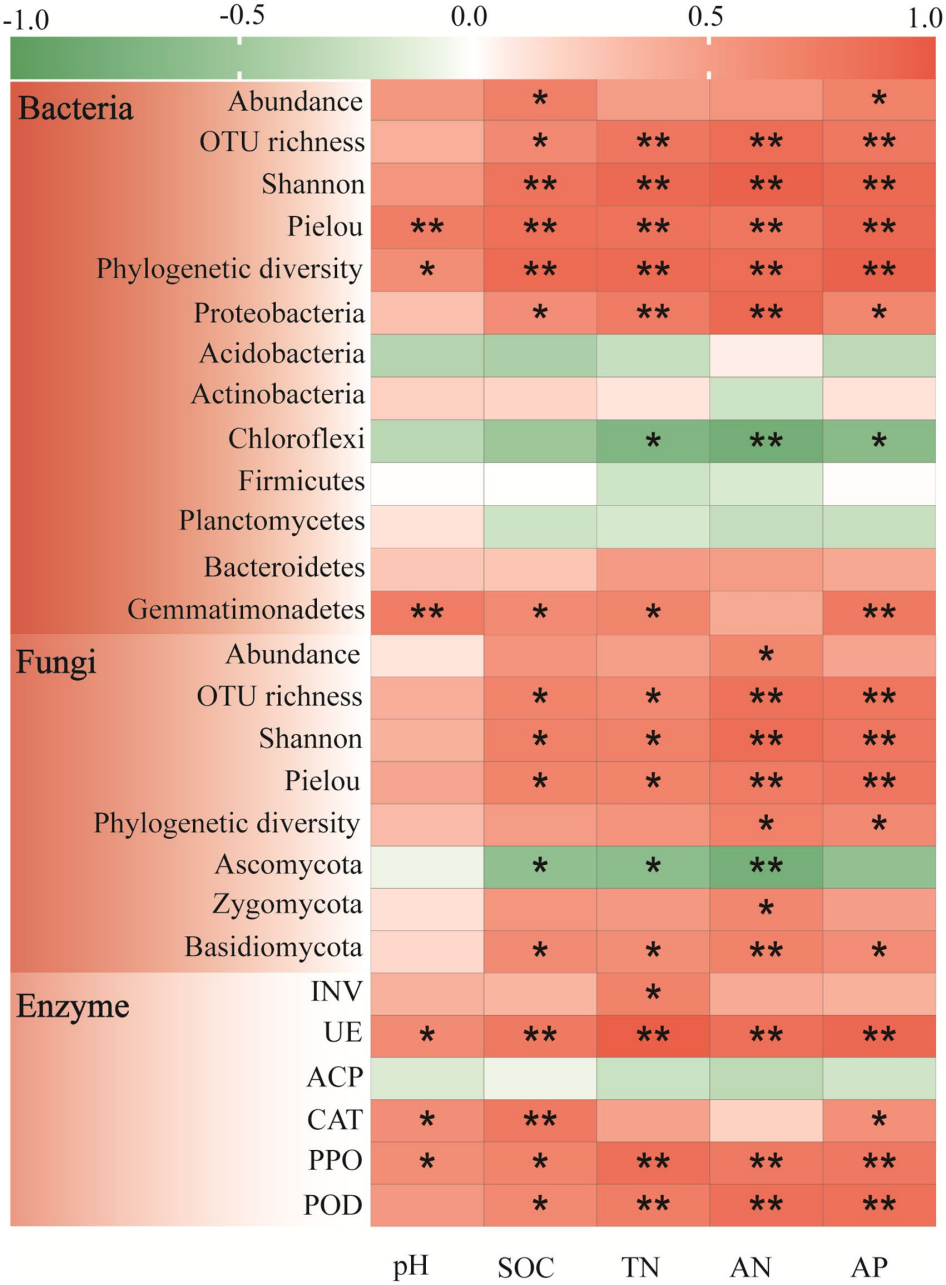
Pearson correlation analysis of soil fertility indexes with microbial community composition (abundance, alpha-diversity and dominant phyla) and enzyme activities in the *Cinnamomum camphora* coppice planting. Red represents a positive correlation, and green represents a negative correlation. (*p < 0.05; **p < 0.01).

Pearson correlation analysis was performed to further characterize the relationships between the soil microbial biomass, alpha-diversity, and dominant genera with soil fertility (Figure 2). The soil bacterial biomass was significantly positive correlated to SOC (*r* = 0.705, *p* < 0.05) and AP (*r* = 0.687, *p* < 0.05). As a whole, the four bacterial alpha-diversity indexes had significant positive correlation with all the soil fertility indexes (*p* < 0.01). For the dominant bacteria phyla, the Proteobacteria was significantly positive correlated with all the soil fertility indexes except pH, and the Gemmatimonadetes had significantly positive correlation with soil pH, SOC, TN and AP. However, the Chloroflexi was significantly negative correlated with soil TN (*r* = −0.701, *p* < 0.05), AN (*r* = −0.795, *p* < 0.01) and AP (*r* = −0.645, *p* < 0.05).

Further analyses illustrated that the soil fungal biomass only had a significant positive correlation with AN (Figure 2; *r* = 0.659, *p* < 0.05). Notably, the fungal OTU richness, Shannon index, and pielou indexes were all significantly positively correlated with all the soil fertility indexes except pH, whereas phylogenetic diversity was only significantly positively correlated with AN (*r* = 0.696, *p* < 0.05) and AP (*r* = 0.632, *p* < 0.05). And the Zygomycota was only significantly positive correlated with AN (*r* = 0.651, *p* < 0.05). Overall, the Basidiomycota was positively correlated with all the soil fertility indexes, while the Ascomycota showed the opposite trend.

Correlation analysis was also detected between soil enzyme activities and soil fertility (Figure 2). In general, the activities of soil UE, PPO, and POD were significantly positive correlated to all the soil fertility indexes. The soil CAT activity had significant positive correlation with pH (*r* = 0.599, *p* < 0.05), SOC (*r* = 0.751, *p* < 0.01), and AP (*r* = 0.584, *p* < 0.05), while the soil INV activity only positive correlated with TN (*r* = 0.677, *p* < 0.05). Nevertheless, the soil ACP activity was slightly negative correlated with all the soil fertility indexes.

### Primary factors for changes in soil microbial community and the enzyme profiling

**Figure 3:**
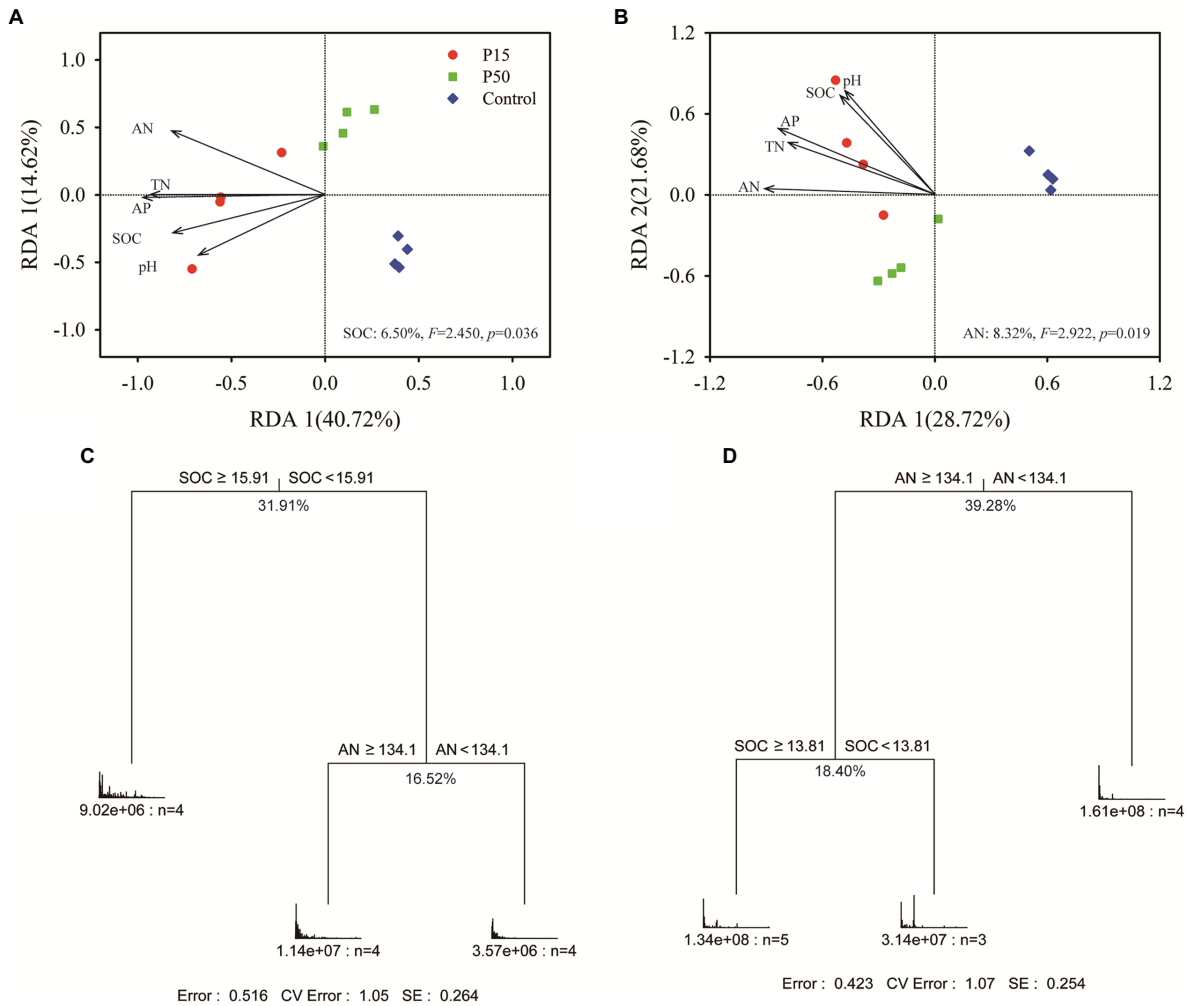
Redundancy analysis (RDA) between the soil bacterial **(A)**, fungal **(B)** community and fertility indexes at the OTU level in the *Cinnamomum camphora* coppice planting. P15, point under the tree canopy; P50, point between trees; Control, point in the abandoned land. Multivariate regression trees (MRT) of soil bacteria **(C)**, fungi **(D)** community composition and fertility indexes. Numbers under the crosses of each split indicate percentages of variance explained by the split.

The CAP analysis showed significant differences in soil microbial community between treatments (Supplementary Figure S4). Remarkably, we found that the soil bacterial community clearly separated (*p* = 0.001) among the three treatments (Supplementary Figure 4A; 43.1% of total variance was explained, *p* = 0.001). ANOSIM tests (Supplementary Table S1) revealed significantly (*p* < 0.05) different soil bacterial community among each other. Mantel test exhibited that soil bacterial community had significant (*p* < 0.01) correlation with all the soil fertility indexes (Supplementary Figure S5). In addition, RDA analysis pointed that the SOC (*F* = 2.450, *p* = 0.036) significantly affected soil bacterial community composition (Figure 3A). Furthermore, the MRT analysis performed that the SOC produced the largest deterministic effects on soil bacterial community variation (31.91%), with a threshold of 15.91 g kg^−1^ (Figure 3C).

Correspondingly, the CAP analysis corroborated obvious differences in soil fungal community (Supplementary Figure S4B, 39.6% of total variance was explained, *p* = 0.001), and the ANOSIM tests indicated there were significantly different (*p <* 0.05) between treatments (Supplementary Table S1). Mantel test (Supplementary Figure S5) further verified that soil fungal community had significant correlation with soil fertility indexes (*p* < 0.05), especially the soil AN and AP (*p* < 0.01). Similarly, RDA analysis suggested that the soil AN (*F* = 2.922, *p* = 0.019) was the primary factor shifting the fungal community composition. And MRT analysis showed that the soil AN produced the largest deterministic effects on soil fungal community variation (39.28%), with a threshold of 134.1 g kg^−1^ (Figure 3D).

In addition, CAP plot demonstrated that the soil enzyme profiling was also clearly segregated (*p* = 0.007) in three different treatments, which explains 50.4% of the variance in the data (Supplementary Figure 4C). ANOSIM tests pointed that the soil enzyme profiling in P15 and P50 were altered significantly (*p* < 0.05) compared with Control, but there was insignificant difference between P15 and P50 (*r* = 0.0625, *p* = 0.318). Unlike the soil microbial community, Mantel test (Supplementary Figure S5) revealed that there were no significant relationships between the soil enzyme profiling and fertility indexes.

### Correlations between soil enzyme activities and microbial community

**Figure 4:**
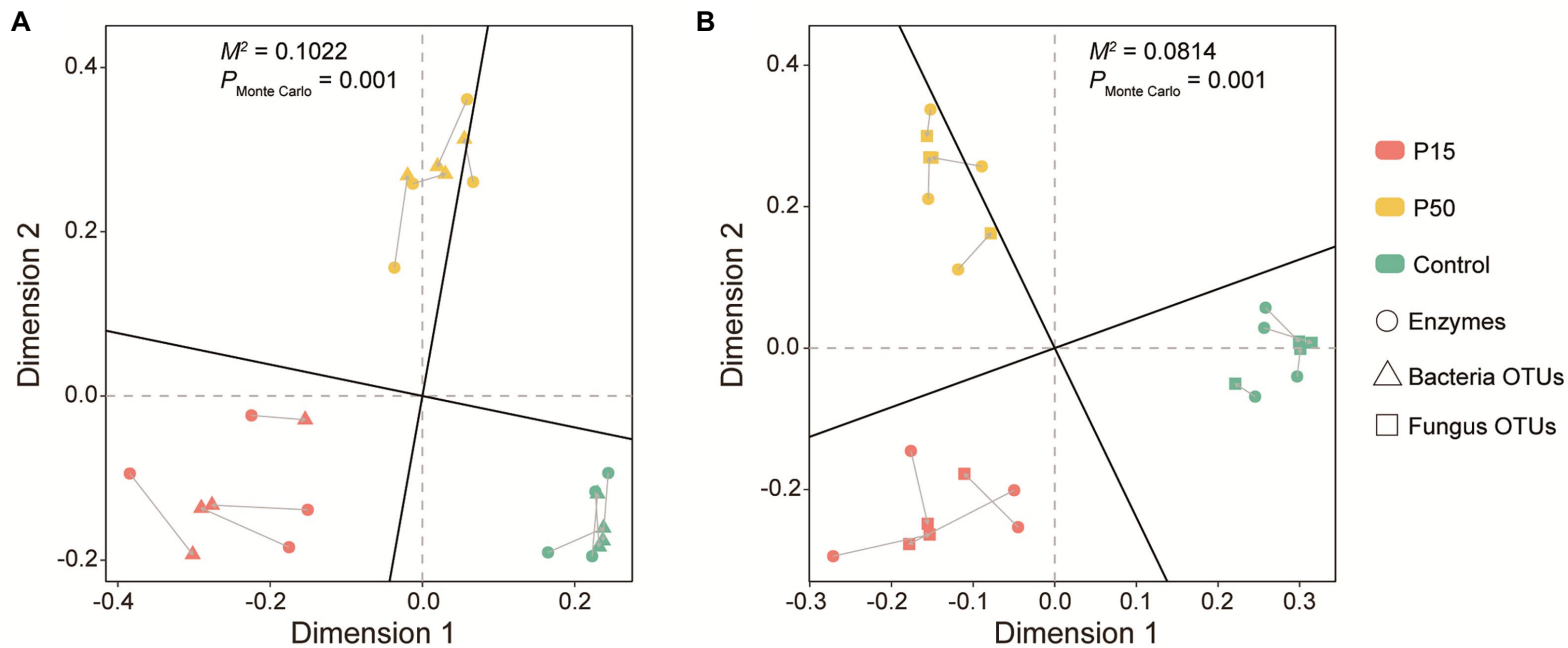
Procrustes analysis between soil bacterial **(A)**, fungal **(B)** community composition and enzyme profiling in the *Cinnamomum camphora* coppice planting. P15, point under the tree canopy; P50, point between trees; Control, point in the abandoned land. The lengths of connecting lines represent the dissimilarities of soil bacterial, fungal community composition and enzyme profiling.

Due to the fact that there had no significant correlation between the soil enzyme profiling and fertility indexes, we further performed our analysis with Procrustes tests to find the linkages between the soil enzyme profiling and microbial community. Procrustes tests corroborated that the soil enzyme profiling had significant correlation with the soil bacterial (Figure 4A, Correlation *r* = 0.9475, *M^2^* = 0.1022, *p* = 0.001) and fungal community (Figure 4B, Correlation *r =* 0.9584, *M^2^* = 0.0814, *p* = 0.001).

**Figure 5:**
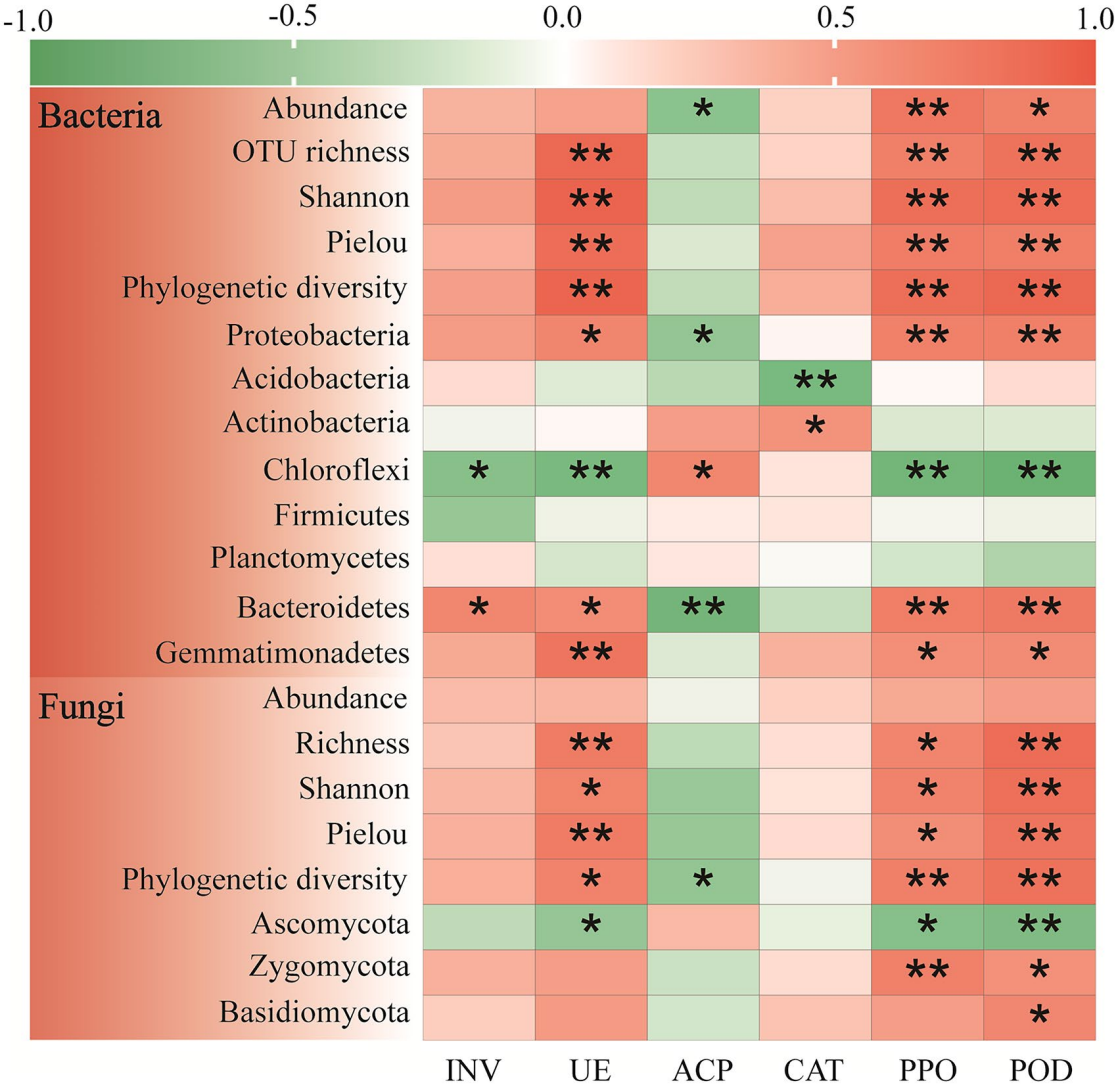
Pearson correlation heat maps between soil bacterial, fungal community composition (abundance, alpha-diversity and dominant phyla) and enzyme profiling in the *Cinnamomum camphora* coppice planting. Red represents a positive correlation, and green represents a negative correlation. (*p < 0.05; **p < 0.01).

Furthermore, relationships between soil enzyme activity and microorganism properties were testified by Pearson correlation analysis (Figure 5). In general, the soil UE, PPO, and POD activities had significantly positive correlation with soil bacterial biomass and alpha diversity, whereas the soil INV and CAT activities had no significant correlation with the above indexes. Notably, soil ACP activity only had significant negative relevance with the bacterial biomass (*r* = −0.635, *p* < 0.05). The soil UE, PPO, and POD activities were significantly positive connected with Proteobacteria, Bacteroidetes and Gemmatimonadetes, but negative connected with Chloroflexi. While the soil ACP activity showed the completely opposite tendency. Meanwhile, the soil INV activity had significantly positive relationship with Bacteroidetes (*r* = 0.665, *p* < 0.05) and significantly negative relationship with Chloroflexi (*r* = −0.665, *p* < 0.05). And the soil CAT activity was significant and positive connected with Actinobacteria (*r* = 0.579, *p* < 0.05) whereas significant and negative connected with Acidobacteria (*r* = −0.773, *p* < 0.01).

Correspondingly, the soil UE, PPO, and POD activities were significant and positive related to the four fungal alpha-diversity indexes, while the soil ACP activity was only significantly negative related to fungal phylogenetic diversity (*r* = −0.596, *p* < 0.05). The soil UE, PPO, and POD activities had significantly negative correlation with Ascomycota. In the meantime, the soil POD activity had significantly positive correlation with Zygomycota (*r* = 0.593, *p* < 0.05) and Basidiomycota (*r* = 0.661, *p* < 0.05), whereas the soil PPO activity only significantly positively correlated with Zygomycota (*r* = 0.713, *p* < 0.01). Remarkably, the soil INV and CAT activities had no significant correlation with all the soil fungal properties.

## Discussion

### Effects of *Cinnamomum camphora* coppice planting on soil fertility

In this study, the *C. camphora* tree was cultivated as coppice and its aerial part were annually cut down to extract the essential oil. This will inevitably take away nutrients from the soil. However, we found that relative to the abandoned land, the point under the tree canopy (P15) substantially increased the soil pH and N, P contents. Firstly, fertilization can compensate for the high quantities of soil nutrients consumed by the *C. camphora* trees (Selvaraj et al., 2017). Secondly, though *C. camphora* coppices were felled during July to September every year, they sprouted and form the canopy rapidly, which can effectively reduce the erosion caused by the concentrated rainfall season (March to July) in south China (Hinojosa et al., 2021; Tang et al., 2021). Moreover, the continuously growing roots of the *C. camphora* tree can promote the activation and transportation of soil mineral elements (Männistö et al., 2016). Earlier study had suggested that the soil nutrients content under the tree canopy was higher than the point outside the tree canopy in the *Artemisia ordosica* Krasch planting (Qi et al., 2010). On account of the “fertility islands” effects that the soil fertility gradually decreased from the inside to the outside under the tree canopy (San Emeterio et al., 2021). Nevertheless, our research revealed that even the point between trees (P50), which was weakly affected by fertilization and tree growth, can somehow enhance the soil nutrients availability (Han et al., 2021). This further corroborated that the *C. camphora* tree planting can improve the soil fertility.

### Effects of *Cinnamomum camphora* coppice planting on soil microbial structure

Soil microorganisms are important regulatory factors in soil organic matter transformation, and they may influence by changes in soil fertility after tree planting (Thébault et al., 2014). Huang et al. (2022) recorded that tree planting enhanced the SOC and TN contents, and they had notable correlation with soil microbial biomass and diversity. A previous study on *Cryptomeria japonica* also verified that the increase of the soil C and N nutrients stimulated the improvement of soil microbial biomass, OTU richness, and Shannon index (Sawada et al., 2021). Likewise, our results proved that the *C. camphora* tree coppice planting enhanced the bacterial, fungal biomass and diversity (Figure 1), and their change was induced by increased soil fertility (Figure 2). One possible reason was that the *C. camphora* tree coppice planting can produce a large amount of soil N, P nutrients and C substratum, which in turn provide more diverse niches available for microorganisms (Pereira et al., 2019; Asigbaase et al., 2021).

Alterations in soil microorganisms after tree planting were closely correlated with their ecological strategies (Fierer et al., 2007). The *C. camphora* coppice planting significantly influenced the soil microbial dominant phyla (Supplementary Figure S2). Proteobacteria was the typical *copiotrophs* (*r*-strategist), which generally exists in high C and N soils (Trivedi et al., 2013). An earlier study indicated that mixed *Eucalyptus grandis* plantings significantly improved soil fertility, and relative abundance of Proteobacteria increased from 14.29 to 27.90% (Zhang et al., 2022). In the present study, relative abundance of Proteobacteria increased significantly by 7.47 and 11.60% in P15 and P50, respectively. This also reflected that the *C. camphora* coppice planting can increase soil fertility. Similarly, Zygomycota and Basidiomycota were also *copiotrophs*, which can form the mycorrhiza with tree roots and use readily available nutrients in a short period (Wang et al., 2017; Zhu et al., 2019). Their relative abundance was also significantly improved in the *C. camphora* coppice planting (Supplementary Figure S2). And the correlation analysis further identified that Proteobacteria, Zygomycota, and Basidiomycota were significantly positively correlated with soil fertility indexes (Figure 2).

On the contrary, Chloroflexi and Ascomycota were the typical *oligotrophs* (*K*-strategist), early studies reported that with increase of soil fertility, relative abundance of Chloroflexi and Ascomycota were decreased (Zhu et al., 2019; Idbella et al., 2022). This further confirmed by our research that relative abundance of Chloroflexi and Ascomycota were significantly negative correlated with soil fertility indexes in general (Figure 2). Additionally, *Ktedonobacter* was the member of Chloroflexi, and *Talaromyces* and *Hyaloscyphaceae* were the members of Ascomycota. STAMP analysis indicated that their relative abundance was declining when soil fertility was improved. And in the low fertile soil, such as P15 *vs* Control, *Ktedonobacter*, *Talaromyces* and *Hyaloscyphaceae* were the significantly different species between treatments (Supplementary Figure S3). In conclusion, the *C. camphora* coppice planting shifted the soil microbial community from *oligotrophs* to *copiotrophs*, and relative abundance of *copiotrophs* microorganisms in P15 were increased from 37.78% (bacteria), 18.09% (fungi) to 53.80% (bacteria), 29.24% (fungi), respectively (Supplementary Table S2).

Various studies have summarized that soil microbial community compositions changed under tree planting. The conversion from natural forest to tree planting had significantly changed the community of soil bacteria (Jiang et al., 2021; Li et al., 2022), and SOC was the primary factor for its change (Zhang et al., 2016). This was consistent with our results, indicating that SOC can provide resources for soil bacterial community and modify its ecological strategy, thus affecting soil bacterial community composition (Qu et al., 2020; Yang et al., 2021a). Additionally, soil fungal community significantly shifted in the *C. camphora* coppice planting (Supplementary Figure S4), and RDA, MRT analysis exhibited that AN produced the largest deterministic effects to them (Figure 3). This had also confirmed by Wang et al. (2021) who recorded that soil fungal community composition had significant positive relation to soil AN content in *Cunninghamia lanceolate* (Lamb.) Hook plantings.

### Effects of *Cinnamomum camphora* coppice planting on soil enzyme activities

Soil enzymes, as the participants in soil organic matter transformation, can also make vital functions in carbon (C), nitrogen (N), and phosphorus (P) cycling of soil ecosystems (Feyissa et al., 2022). our results declared the *C. camphora* coppice planting significantly enhanced the soil INV, UE, PPO and POD activities (Table 2). And correlation analysis suggested that soil enzyme activities except soil ACP showed positive relationship with soil pH and fertility indexes in general (Figure 2). Many previous studies pointed out that soil INV and UE activities were involved in soil C and N transformation, respectively (Boughattas et al., 2019; Zhu et al., 2019). Wang et al. (2018) acknowledge that *Camellia sinensis* L. planting increased soil C, N concentration, and soil INV, UE activities were also improved with the stand age. Moreover, soil PPO and POD were involved in the oxidation and degradation of soil lignin and SOM (Sinsabaugh, 2010), and their increase illustrated that *C. camphora* coppice planting can improve soil nutrients availability. Nevertheless, in the present study, soil ACP activity under the tree canopy (P15) and between the trees (P50) was significantly decreased. Though the soil ACP is involved in the decomposition of soil organic phosphorus to promote its metabolic turnover and reuse efficiency, the increasing soil available phosphorus content will reduce the ACP activity (Tran et al., 2010). In other words, high nutrient availability may inhibit soil ACP activity (Janes-Bassett et al., 2022), and we also found that there were negative correlations between soil ACP activity and soil fertility (Figure 2). And STAMP analysis further revealed that soil ACP activity was higher in the low fertility soil (Supplementary Figure S3).

Our work corroborated that soil enzyme profiling was weakly (*p* > 0.05) affected by soil fertility (Supplementary Figure S5), while they were highly connected with soil bacterial and fungal community (Figure 4). Further correlation analysis identified that soil UE, PPO, and POD activity was significantly positively correlated with soil microbial biomass and diversity (Figure 5). Earlier studies declared that soil microbial biomass, diversity (e.g., OTUs, Shannon) and enzyme activities (e.g., UE and POD) were all significantly increased after afforestation. (Thoms et al., 2010; Huang et al., 2022). For one thing, soil microorganisms have their unique ecological functions, and the increasing soil microbial diversity may enhance the quantities of specific-species involved in soil nutrient cycling (Weand et al., 2010). For another, the enhancement of soil microbial biomass will promote the secretion of soil extracellular enzymes (van der Heijden et al., 2008). As for the dominant microbial phylum, we found that soil UE, PPO, and POD activities had significantly positive correlation with *copiotrophs* (e.g., Proteobacteria, Bacteroidetes and Gemmatimonadetes), while they had significantly negative correlation with *oligotrophs* (e.g., Chloroflexi and Ascomycota). As soil UE, PPO and POD were conducive to recalcitrant carbon decomposition, their increase may signify the enhancement of soil nutrient supplying capacity (Yang et al., 2021b). This also confirmed that the *C. camphora* coppice planting can improve soil fertility. Conversely, soil ACP activity was significantly positive connected with Chloroflexi (*oligotrophs*), whereas significantly negatively related to Proteobacteria (*copiotrophs*). These findings suggested that Chloroflexi have more survival advantages in low soil fertility (abandoned land), which may promote the secretion of soil ACP (Kaiser et al., 2010; Gong et al., 2019).

## Conclusion

The present study indicated that the *C. camphora* coppice planting significantly increased the soil fertility, and therefore cause a corresponding alteration in soil microbial community composition. Compared with the abandoned land, the microbial biomass and diversity were significantly higher under the tree canopy (P15) and between the trees (P50). The *C. camphora* coppice planting shifted the soil microbial community from *oligotrophs* (*K*-strategist) to *copiotrophs* (*r*-strategist). SOC and AN were the primary factors that drive changes in soil bacterial, fungal community composition, respectively. In addition, soil UE, PPO, and POD activities were significantly increased, while soil ACP activity was significantly decreased. And soil bacterial and fungal community significantly influenced the soil enzyme profiling. Soil UE, PPO, and POD activities were significantly positive correlated with *copiotrophic* microorganisms, while significantly negative correlated with *oligotrophic* microorganisms, and soil ACP activity show the opposite trend. Overall, the *C. camphora* coppice planting led to significant changes in soil fertility, microbial community structure, and enzyme activities, at which the point under the tree canopy (P15) had more significant effects than the point between trees (P50). This work can provide a theoretical basis for soil sustainable utilization and productive management of *C. camphora* coppice plantation in subtropical China.

## Data availability statement

The original contributions presented in the study are included in the article/Supplementary material, further inquiries can be directed to the corresponding authors.

## Author contributions

LS, JieZ, and YL contributed to conception and design of the study. LS, JieZ, JiaZ, XL, YG, HS, HL, and YL completed the field sampling. LS, JieZ, CX, ZX, TZ, and YL performed the statistical analysis. LS, JieZ, and YL wrote the manuscript. LS, JieZ, JiaZ, XL, CX, ZX, TZ, and YL contributed to the revision of manuscript. All authors contributed to the article and approved the submitted version.

## Funding

This study is supported by Jiangxi Provincial Science and Technology Program (20204BCJL23046, 20181ACF60002), the National Natural Science Foundation of China (32060333, 52269013), and the National Key Research and Development Program of China (no. 2021YFD1901201-05).

## Conflict of interest

The authors declare that the research was conducted in the absence of any commercial or financial relationships that could be construed as a potential conflict of interest.

## Publisher’s note

All claims expressed in this article are solely those of the authors and do not necessarily represent those of their affiliated organizations, or those of the publisher, the editors and the reviewers. Any product that may be evaluated in this article, or claim that may be made by its manufacturer, is not guaranteed or endorsed by the publisher.
