# Supplementary material for: Effects of Cinnamomum camphora coppice planting on soil fertility, microbial community structure and enzyme activity in subtropical China
Source: Front Microbiol. 2023 Feb 3;14:1104077. doi: 10.3389/fmicb.2023.1104077 (PMC9936984; doi:10.3389/fmicb.2023.1104077)
Supplement: Supplementary file 1 [file Data_Sheet_1.docx]

**Table S1** Difference of bacterial, fungal community and enzyme profiling based on the similarity test of ANOSIM.

| Treatment | Bacteria | | Fungi | | Enzyme | |
| --- | --- | --- | --- | --- | --- | --- |
|  | *r* | *p* | *r* | *p* | *r* | *p* |
| P15 vs P50 | **0.8021** | **0.030** | **0.6042** | **0.029** | 0.0625 | 0.318 |
| P15vs Control | **0.8854** | **0.029** | **0.7604** | **0.036** | **0.5312** | **0.032** |
| P50vs Control | **1.0000** | **0.023** | **0.8854** | **0.027** | **0.9167** | **0.023** |

P15, point under the tree canopy; P50, point between trees; Control, point in the abandoned land. Significant values shown in bold.

**Table S2** Relative abundance of Oligotrophic and Copiotrophic phyla in soil bacterial and fungal community in the *Cinnamomum camphora* coppice planting.

| Treatment | Bacteria | | Fungi | |
| --- | --- | --- | --- | --- |
|  | *Oligotrophic* (%) | *Copiotrophic* (%) | *oligotrophic* (%) | *copiotrophic* (%) |
| P15 | 42.76±0.71c | 53.80±0.44a | 44.7±14.31b | 29.24±4.77a |
| P50 | 48.35±3.61b | 48.53±3.99b | 56.33±9.02ab | 24.37±6.29ab |
| Control | 58.23±1.69a | 37.48±1.45c | 69.15±5.53a | 18.09±7.73b |

P15, point under the tree canopy; P50, point between trees; Control, point in the abandoned land. Values (mean ± S.D.) in the same column followed by different letters are significantly different from Duncan's HSD comparisons (*p* < 0.05)


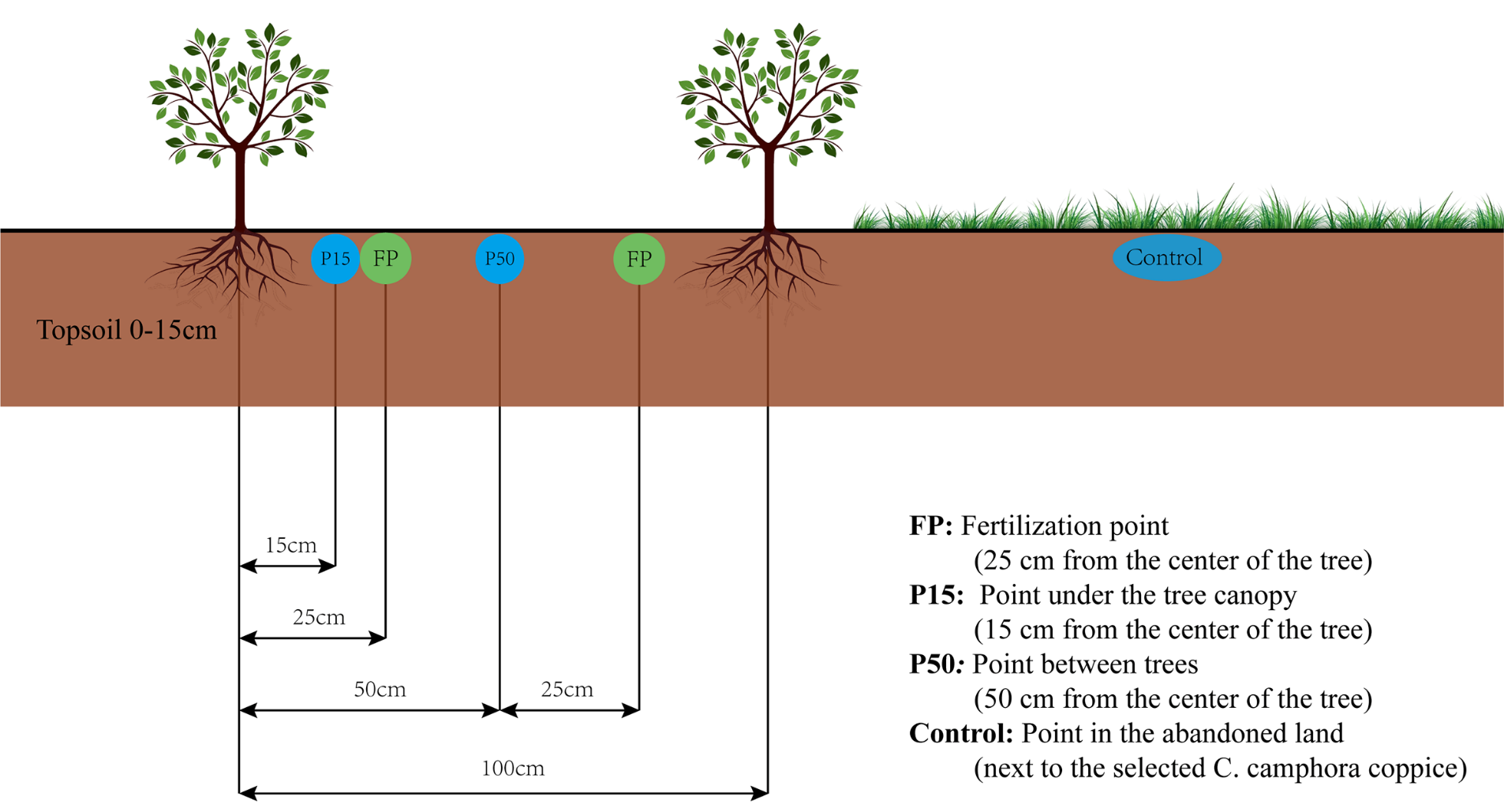


**Figure S1** Experiment layout with treatment details.


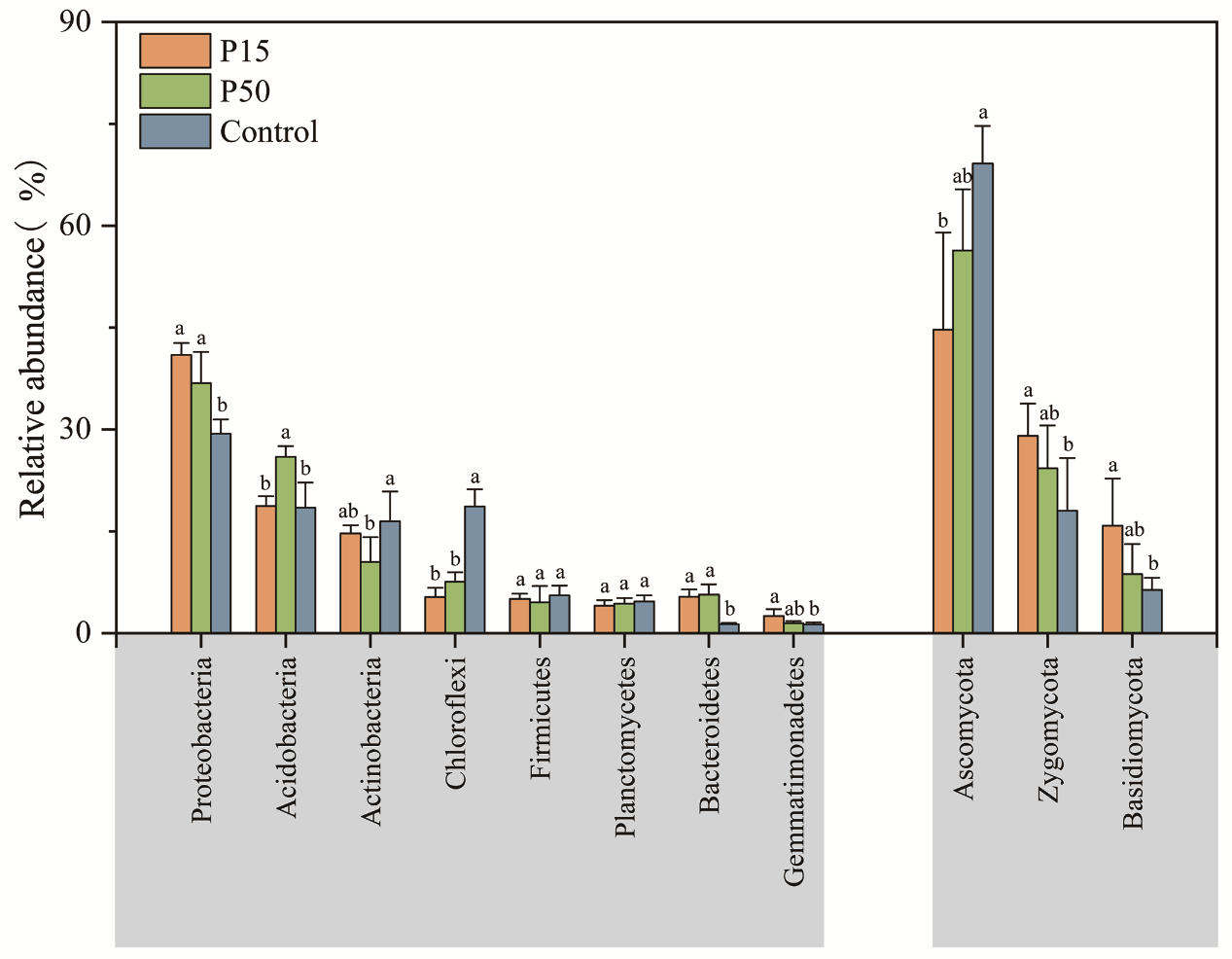


**Figure S2** The relative abundance of bacterial, fungal major phyla (>1%) under different treatments in the *Cinnamomum camphora* coppice planting. P15, point under the tree canopy; P50, point between trees; Control, point in the abandoned land. Bars represent mean; error bars denote standard deviation. Letters above bars represent differences from Duncan's HSD comparisons (*p* < 0.05).


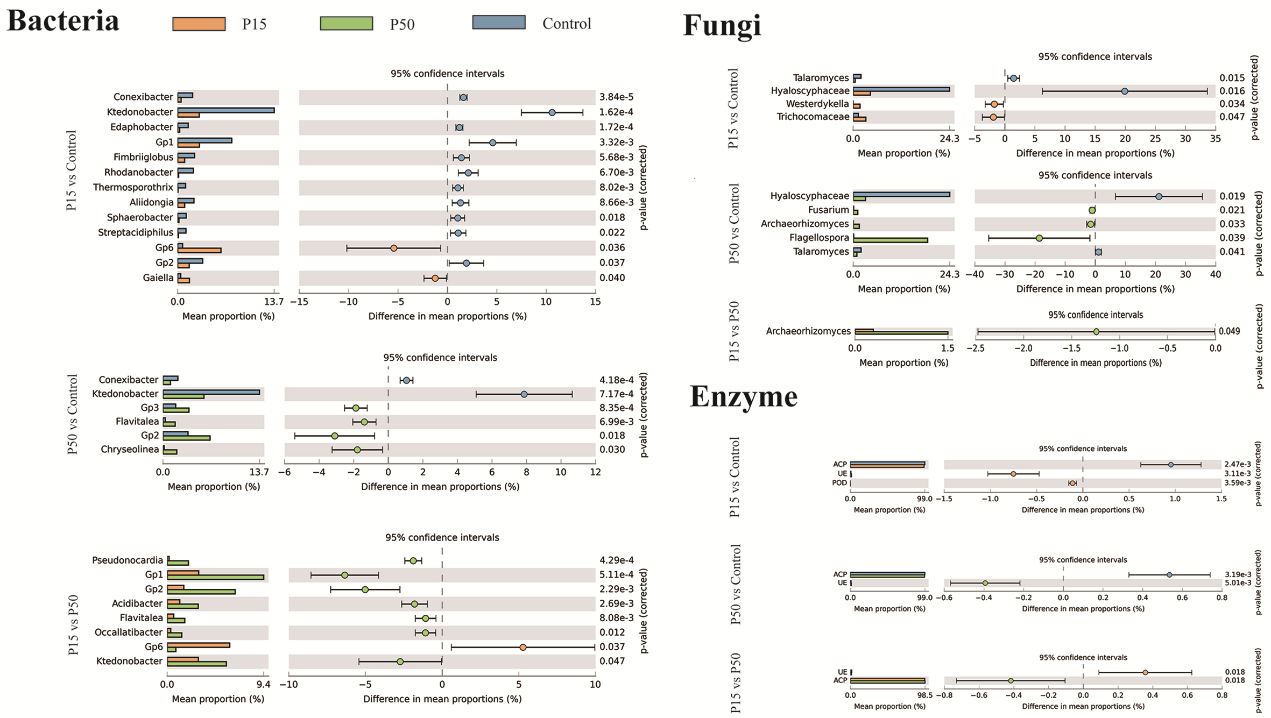


**Figure S3** Significant difference in bacterial, fungal genera and enzyme activities between treatments based on statistical analysis of metagenomic profile in the *Cinnamomum camphora* coppice planting. P15, point under the tree canopy; P50, point between trees; Control, point in the abandoned land.


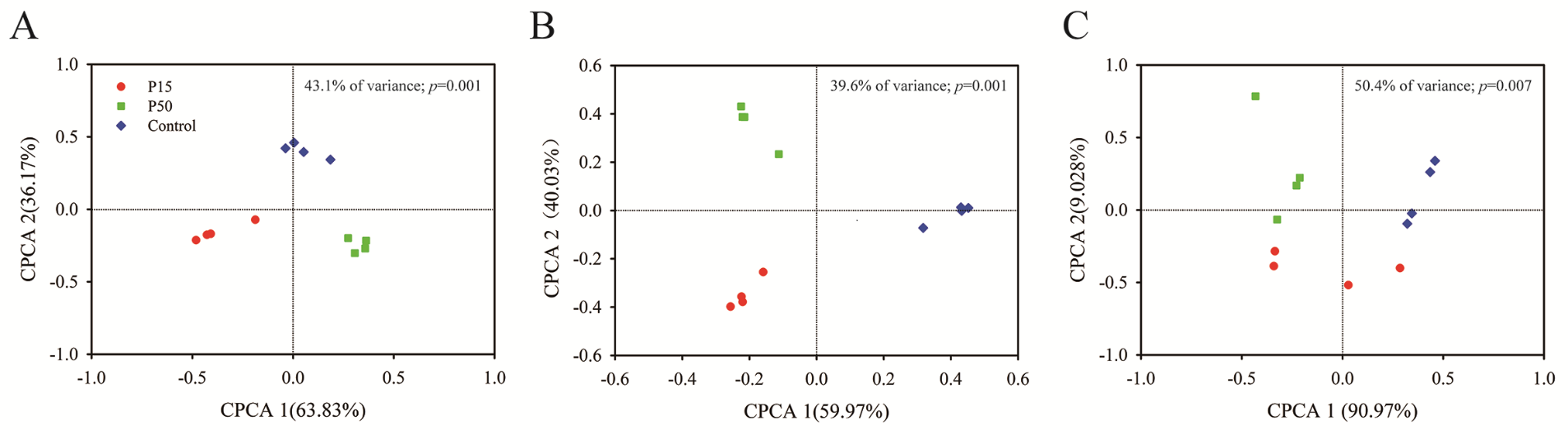


**Figure S4** Constrained principal coordination analysis (CAP) of the bacteria (A), fungi (B) community (based on OTU level) and enzyme profiling (C) based on Bray-Curtis distances in the *Cinnamomum camphora* coppice planting. P15, point under the tree canopy; P50, point between trees; Control, point in the abandoned land. Different shapes and colors represented different treatments. The variation explained by the CAP axes was listed in parentheses.


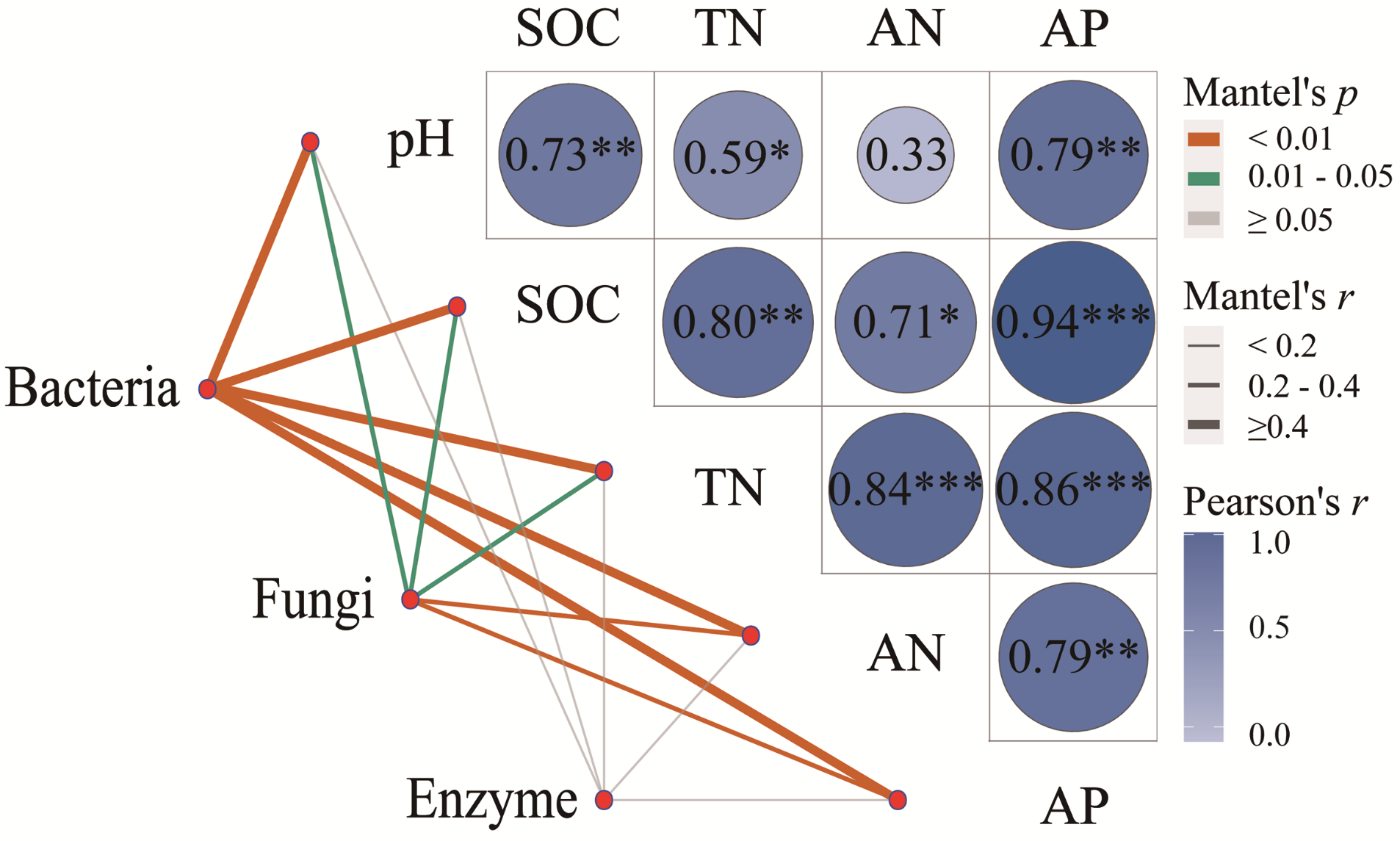


**Figure S5** Pearson correlation heat maps between soil fertility indexes are shown, with a color gradient denoted Pearson's correlation coefficient. Soil bacterial, fungal community composition and enzyme profiling were linked to each fertility indexes by Mantel tests in the *Cinnamomum camphora* coppice planting. Edge width corresponds to the Mantel’s *r* statistic for the corresponding distance correlations, and edge color denotes the statistical significance.
